# Amphibian and reptile biodiversity in the semi-arid region of the municipality of Nopala de Villagrán, Hidalgo, Mexico

**DOI:** 10.7717/peerj.4202

**Published:** 2018-01-04

**Authors:** Andrea J. Roth-Monzón, Andrés Alberto Mendoza-Hernández, Oscar Flores-Villela

**Affiliations:** 1 Departamento de Biología Evolutiva, Facultad de Ciencias, Universidad Nacional Autónoma de México, Ciudad de Mexico, Mexico; 2 Current affiliation: Evolutionary Ecology Laboratories, Department of Biology, Brigham Young University, Provo, UT, United States of America

**Keywords:** Seasonality, Conservation, Biodiversity, Semi-arid regions, Xeric-scrub, Microhabitat, Amphibians, Reptiles

## Abstract

Current global changes are putting both biodiversity and the processes that depend on it at risk. This is especially true for semi-arid regions and the flagship groups that inhabit them, such as amphibians and reptiles. Semi-arid regions are often thought to have lower biodiversity and thus have been overlooked, resulting in the underestimation of their biological richness. Therefore, the aim of this study was to conduct an inventory of amphibians and reptiles in the semi-arid municipality of Nopala de Villagrán, Mexico, and analyze its biodiversity in relation to the seasons, vegetation and microhabitat. During a year of fieldwork, we found 24 species in the area, most of them of low abundance, and one of which was recorded for the first time for the state of Hidalgo. We documented five amphibian species and 19 reptile species. We also found that observed species richness was higher in the rainy season and in xeric scrub vegetation, although only the season differences were significant according to rarefaction curves. Our findings highlight the importance of seasonality and vegetation type for the species that inhabit this semi-arid region. This study broadens our understanding of the importance of semi-arid regions and, by extension, that of other areas with similar characteristics.

## Introduction

Current global changes are affecting both human societies and biodiversity assemblages, putting many of the ecosystem processes that depend upon biodiversity at risk (Díaz et al., 2006; Ceballos et al., 2015). Biodiversity provides several benefits to ecosystems including ecological efficiency, ecosystem stability and ecosystem productivity, among others (Cardinale et al., 2012). Thus, it is important to know, conserve and maintain biodiversity before it is lost (Cardinale et al., 2012). Although in recent years greater efforts have been made to document and protect biodiversity, there is still the need to continue such efforts (Purvis & Hector, 2000).

Priority should be given to important species and regions that have been recognized as conservation flagships or are understudied regions for biodiversity. Drylands, such as deserts, semi-arid regions and arid regions, comprise one natural biome that is at risk (Davies et al., 2012; Bastin et al., 2017). The vulnerability of these regions is a result not only of human development that reduces their area, but also of climate change and land use practices that may lead to desertification (Davies et al., 2012). Furthermore, drylands have been largely neglected in the literature (Bastin et al., 2017; Durant et al., 2012). Because conservation policies are species driven (Agapow et al., 2004) efforts to maintain and study biodiversity focus on “hot spots” and tropical forest that maximize the number of species conserved (Myers et al., 2000). However, dryland biomes cover approximately 41.5% of the Earth, can contain some of the most threatened species and can house an important number of endemic species (Flores-Villela & Gerez, 1994; Davies et al., 2012; Bastin et al., 2017; Durant et al., 2012).

Drylands are also of great importance for certain taxonomic groups that are found more commonly in these types of environments (Davies et al., 2012). Reptile species are known to be associated with arid climates and niches (Pie, Campos & Duran, 2017; Davies et al., 2012). The study of these environments can be particularly important for such taxonomic groups. About 60% of Mexico is comprised of semi-arid regions (Rzedowski, 1996), a type of dryland, and the country has the second-highest biodiversity of amphibians and reptiles species in the world (Flores-Villela & Canseco-Márquez, 2004; Llorente-Bousquets & Ocegueda, 2008). Therefore, it is important to document the species of amphibian and reptiles in such semi-arid regions, especially in light of their recent decline due to degradation of their natural habitats (Velázquez, Mas & Palacio-Prieto, 2010).

The municipality of Nopala de Villagrán is a semi-arid region (as defined by the aridity index, see methods) in the state of Hidalgo that is located in the Trans-Mexican Volcanic Belt, a highly endemic area where 78.3% of the species of amphibians and reptiles that occur there are endemic (Flores-Villela & Canseco-Márquez, 2007). Furthermore, the number of municipal-level natural reserves in Hidalgo has been increased from 29 to 33, including one in the municipality of Nopala that covers 1,753.7 ha of an area known as Cerro Nopala-Hualtepec (COEDEH, 2009). However, because municipal-level natural reserves were only recently instituted in Mexico, most lack full legal accreditation and management programs based on species inventories (Bezaury-Creel & Gutiérrez Carbonell, 2009), making it important to determine exactly what biodiversity is being preserved in these areas.

Although some biological inventories that assess amphibian and reptile biodiversity have already been carried out in Hidalgo, they have been conducted mainly in the northern areas due to the natural reserves located there (CONANP, 2003; CONANP, 2006; CONANP, 2007; COEDEH, 2009; Ramírez-Bautista et al., 2014). The municipality of Nopala de Villagrán is located in the southwestern portion of the state, in which only one previous study has been carried out (Gómez Mendoza, 2007).

The aim of this study was to document the biodiversity of amphibians and reptiles in the semi-arid municipality of Nopala de Villagrán, Hidalgo, and to describe the current patterns of biodiversity with respect to the type of vegetation, seasonality and microhabitat. Identifying and describing the distribution of these amphibians and reptiles will make it possible to inform conservation decisions and management strategies for this and other similar regions in Mexico.

## Materials and Methods

**Figure 1:**
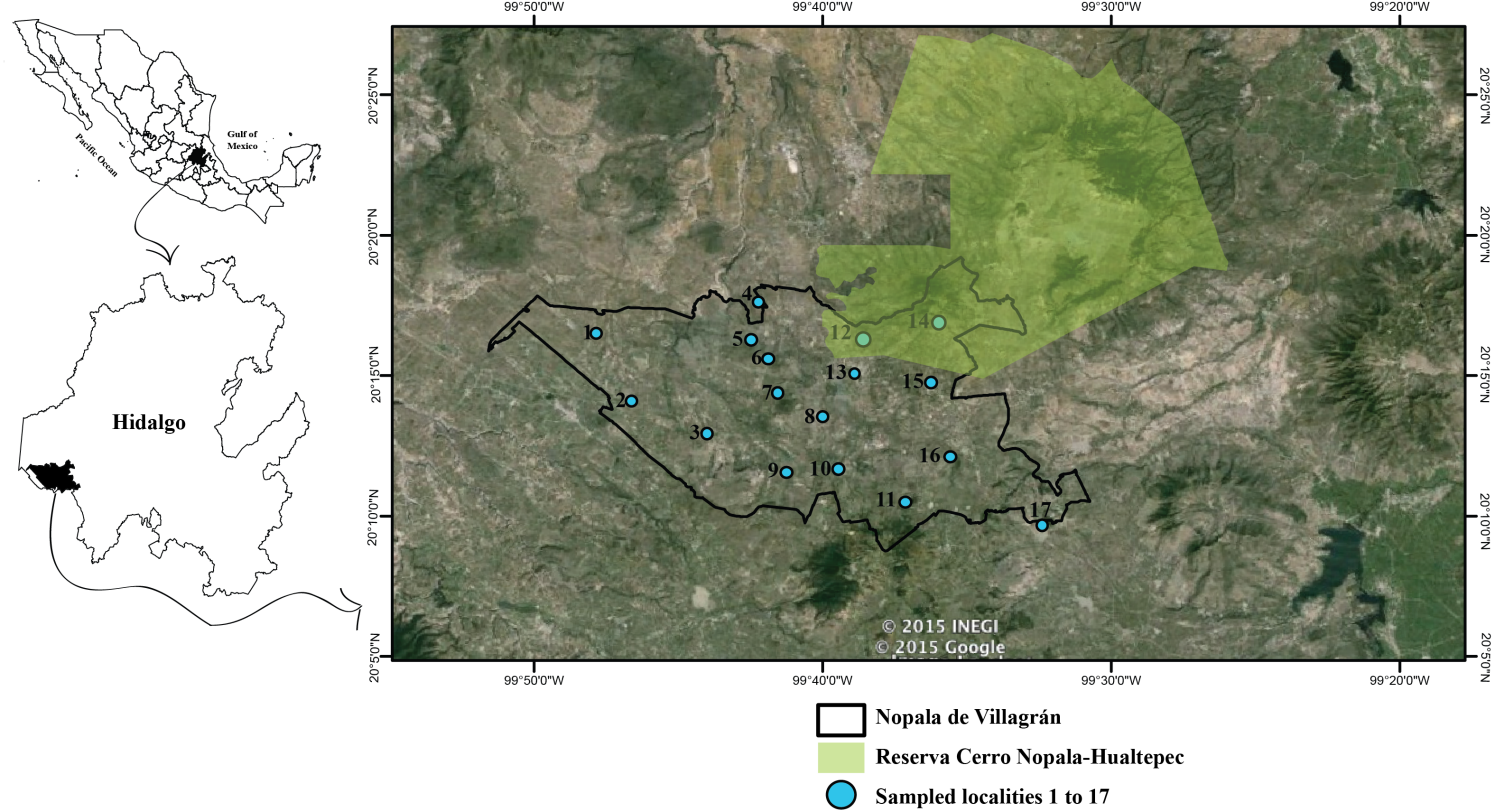
Location of the municipality of Nopala de Villagrán, in the state of Hidalgo, Mexico. Blue dots with numbers show the location of the sites surveyed in this study. Black outline indicates the municipality of Nopala de Villagrán. Green area is the Reserva Cerro Nopala-Hualtepec. Image Spot January 2015 Google Maps and location of the Cerro Nopala-Hualtepec taken from Rivas et al. (2009), polygon obtained from http://www.conacyt.gob.mx/cibiogem/index.php/anpl/hidalgo.

Our study was designed to determine the diversity of amphibians and reptiles in the municipality of Nopala de Villagrán in the southwestern part of the state of Hidalgo, Mexico (20°18′, 20°09N; 99°31′, 99°51W; INEGI, 2002), at an elevation of 2,400 m a.s.l. (Fig. 1). The municipality is a semi-arid region as determined by the aridity index, i.e., the ratio of mean annual precipitation to annual global terrestrial precipitation (Ponce, Pandey & Ercan, 2000), as its index falls between 0.25 and 0.5.

**Figure 2:**
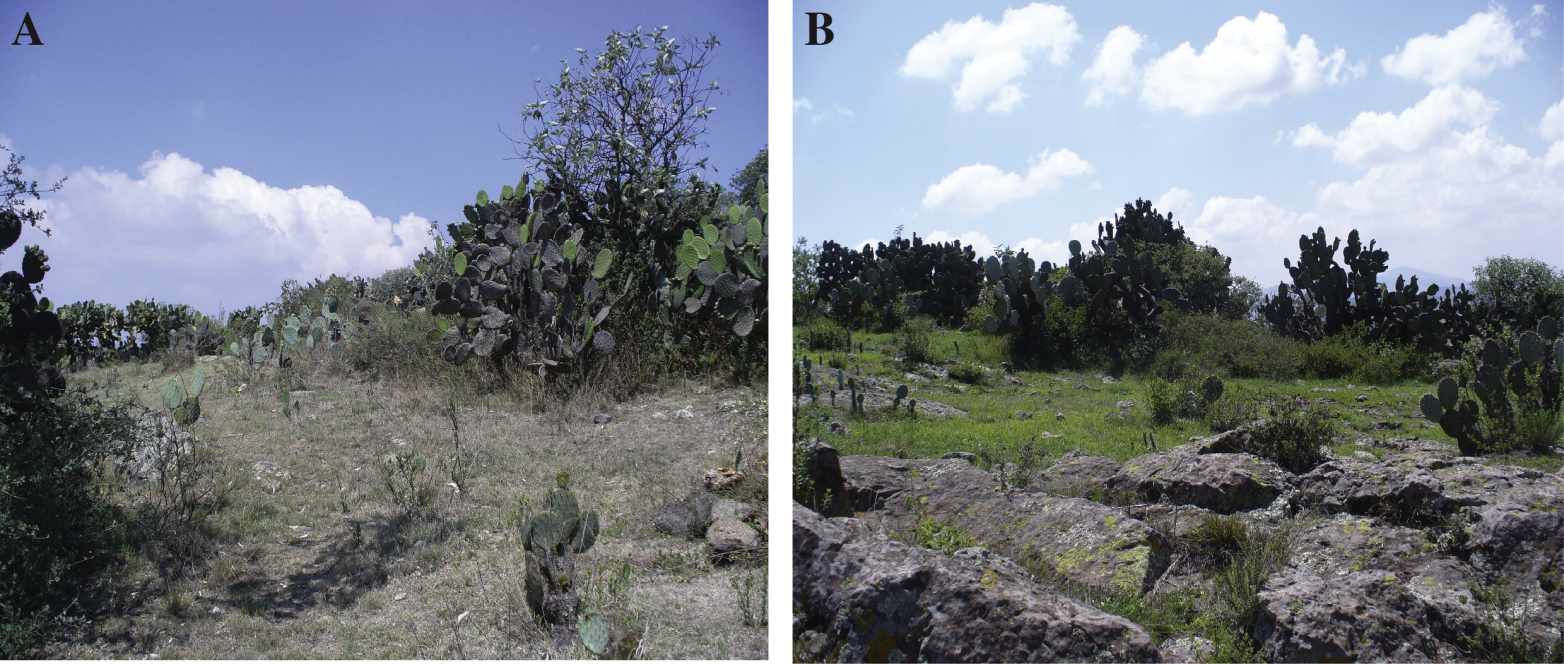
Difference in xeric scrub vegetation in the dry and rainy season. Photograph (A) is locality 16, Cerro Maravillas during the dry season. Photograph (B) is the same locality during the rainy season. Photographs by A.A. Mendoza-Hernández.

Nopala de Villagran is a relatively small municipality with 342.305 km^2^ and a population with approximately 15,666, with the biggest towns with populations <2,500. This is a rural area that is economically supported by farming, the main crops in the municipality are, in order of importance: corn (*Zea mays*), beans, forage oats and agave (INEGI , 2013). The municipality is located in the biogeographical sub-province of the Plains and Highlands of Querétaro and Hidalgo, a sub-providence situated on the Trans-Mexican Volcanic Belt. The climate in the area is temperate subhumid, with a dry season from December to May and a wet season from June to November. The mean annual temperature is 14.7 °C, with a typical minima of 12.8 °C and a maxima of 17.3 °C, and rainfall is 615 mm (INEGI, 2002), making it a semi-arid region (Schlesinger et al., 1990; Ponce, Pandey & Ercan, 2000). There is no permanent surface water or streams in the area and sinkholes depend only on rainfall (Camacho-Camacho, 2005), so there are differences in the vegetation due to the seasonality of the area that create differences in humidity (Fig. 2).

We conducted field surveys (non-selective searches and visual surveys; Crump & Scott, 1994) monthly for a year from March 2008 to February 2009 with the permission of the Secretaría de Medio Ambiente y Recursos Naturales (FAUT-0015). This allowed us to examined temporal variation, as both precipitation and temperature vary throughout the year in the municipality that can affect the distribution and activity of amphibians and reptiles. We surveyed a total of 17 localities in order to represent most of the municipality (Fig. 1). All localities were surveyed at least twice and as broadly as possible. For four days per month, three people spent at least eight hours per day conducting surveys. Active searches where focused on types of habitats and microhabitats preferred by amphibians and reptiles in order to find the most possible. All microhabitats (e.g., under rocks, under vegetation, on rocks, on trees and bushes) were searched for amphibians and reptiles. Likewise both morning, afternoon and nighttime searches were conducted to cover different periods through the day. The 17 localities were used as a reference during fieldwork, but searches were not constrained to a transect or quadrant, as the focus was to cover the municipality as a whole and search exhaustively to obtain the most complete species list possible. We covered on average 124 ha for each locality in each sampling trip, with a lower range of 60 ha and an upper of 304 ha covered in a single visit.

All specimens collected or observed inside the municipality for which proper identification to the species level was possible were included on our species list. Representative voucher specimens were collected for each species, identified to the species level and deposited in the “Museo de Zoología Alfonso L. Herrera” collection at the Facultad de Ciencias at the Universidad Nacional Autónoma de México (MZFC, UNAM; Appendix S1). Taxonomic names follow “the amphibian species of the world” (Frost, 2017) for amphibians and Flores-Villela & Canseco-Márquez (2004) for reptiles.

A species accumulation curve was plotted based on the monthly increments in the number of species collected. To assess sampling effort, the model that best fits the data and the expected number of species occurring in the area were calculated using the Species Accumulation program (Días-Fránces & Soberón, 2005). The models predict the expected number of species that could be encountered in the municipality if sampling effort was increased, using the occurrence of species in the municipality as a proxy for the sampling effort.

For each specimen collected and observed we recorded data regarding: (1) vegetation, based on the three types of vegetation found in the municipality (xeric scrub, oak forest and croplands: mostly corn and agave); (2) season (rainy and dry); and (3) microhabitat, based on nine categories modified from Duellman (1965) and Vargas-Santamaría & Flores-Villela (2006). These microhabitat categories were terrestrial (on the ground), arboreal (on trees or vegetation above 1.5 m), scrubby (on trees or vegetation below 1.5 m), riparian (near streams or water), aquatic (in water), saxicolous (around rocks), human areas (on human-related objects), fossorial (buried), and decaying trunks (under fallen tree trunks).

To test for differences in species richness for each taxonomic group (amphibians and reptiles) by vegetation (xeric scrub, oak forest and croplands) and seasonality (dry and rainy) we performed a sample size rarefaction curve (Chao & Jost, 2012; Chao et al., 2014). This approach allows us to compare species richness even when differences in sampling effort occur, as it is possible to extrapolate the smallest samples and compare species richness estimates at equal sample coverage by using abundance data as a surrogate for sampling effort. As suggested by Chao et al. (2014) rarefaction curves were extrapolated to double the abundance of the smallest sample, but considering the abundance of the highest sample that is being compared (Chao et al., 2014). Therefore, the extrapolation was extended to the highest abundance sample possible in each rarefaction curve, whether it was doubling the abundance of the smallest sample or using the abundance of the highest sample being compared. We conducted the rarefaction curves using the R package iNEXT (Hsieh, Ma & Chao, 2016), with a bootstrap of 300 replicates (as suggested by Chao et al., 2014) to estimate 95% confidence intervals. We compared the vegetation preference of the species of Nopala that are endemic to Mexico with those that are not, using the Webb index (Webb, 1984; Ochoa-Ochoa & Flores-Villela, 2006), where a value between 0 and 1 indicates fewer endemic species than non-endemic species, a value of 1 indicates the same number of endemic and non endemic species and a value higher than 1 indicates more endemic species than non-endemic species. This allowed us to determine which vegetation type had the greatest proportion of endemic species for the area under study. We also compared vegetation similarity by using the Chao Jaccard similarity index (Chao et al., 2005) for all possible pairs of the three vegetation types in the municipality. We chose this index as it is more robust to unequal samples (Chao et al., 2005), which are likely in our study since priority was given to sampling most of the municipality, in which the distribution of the types of vegetation is unequal. The Chao Jaccard similarity index was calculated using the package SpadeR (Chao, Ma & Hsieh, 2015).

We made rank abundance curves in the package BiodiversityR (Kindt & Coe, 2005) for both amphibians and reptiles separately among vegetation types and season. We plotted the relative abundance on a logarithmic scale against the rank order of the species from most to least abundant. We then visually assessed the rank abundance curves to determine the pattern of dominance by vegetation and season, as well as the differences in the abundance pattern present at each vegetation type and season. All statistical analyses were conducted using the R package (R Core Team, 2013).

## Results

We recorded 24 species (five amphibians and 19 reptiles) in the municipality of Nopala de Villagrán. The amphibians are grouped into four families and four genera, and the reptiles in six families and ten genera (Table 1). Among these species, we found one snake that had not been previously recorded for the state of Hidalgo, until we conducted this study: *Lampropeltis ruthveni* (Roth-Monzón Flores-Villela, Mendoza-Hernández & Flores-Villela, 2011). The snake *L. ruthveni* was, however, known to inhabit the neighboring states of Michoacán, Jalisco and Querétaro (Garstka, 1982; Mara, 1995). None of the species found are considered endemic to the Trans-Mexican Volcanic Belt, but 12 species are endemic to Mexico (two amphibians and 10 reptiles; Table 1; Flores-Villela & Canseco-Márquez, 2007). Furthermore, all of the species found are considered native to Mexico (IUCN, 2017). At Nopala de Villagrán, we documented 13.11% of the amphibian and reptile species reported for Hidalgo based on the list by Ramírez-Bautista et al. (2014). Thirteen reptile species collected fall into two of the risk categories established by Mexican federal law, with eight receiving special protection and five being classified as Endangered (SEMARNAT, 2010; Table 1). All the species present in the municipality have been assessed by the IUCN Red list. Most are considered Least Concern (21 species) and only three species fall in a different risk category (Table 1; IUCN, 2017).

**Table 1:** List of species in the municipality of Nopala de Villagrán, Hidalgo, indicating which are endemic and their protection category according to both the Mexican federal law (NOM-059-SEMARNAT, 2010) and the IUCN Red list (IUCN, 2017).

Class			**Protection category**		
Order	Family	Species	Mexican protection	IUCN	Endemic
**Amphibia**	Eleutherodactylidae	*Eleutherodactylus verrucipes*	Pr	VU	E
Anura	Hylidae	*Dryophytes eximius*		LC	
		*Dryophytes arenicolor*		LC	
	Ranidae	*Lithobates montezumae*	Pr	LC	E
	Scaphiopodidae	*Spea multiplicata*		LC	
**Reptilia**	Phrynosomatidae	*Sceloporus microlepidotus*	Pr	LC	E
Squamata		*Sceloporus mucronatus*		LC	E
		*Sceloporus scalaris*		LC	
		*Sceloporus spinosus*		LC	E
		*Sceloporus torquatus*		LC	E
	Teiidae	*Aspidoscelis gularis*		LC	
	Colubridae	*Coluber schotti*		LC	
		*Conopsis lineata*		LC	E
		*Conopsis nasus*		LC	E
		*Lampropeltis ruthveni*	A	NT	E
		*Pituophis deppei*	A	LC	
		*Salvadora bairdi*	Pr	LC	E
	Natricidae	*Thamnophis cyrtopsis*	A	LC	
		*Thamnophis eques*	A	LC	
		*Thamnophis melanogaster*	A	EN	
	Viperidae	*Crotalus aquilus*	Pr	LC	E
		*Crotalus molossus*	Pr	LC	
Testudines	Kinosternidae	*Kinosternon hirtipes*	Pr	LC	
		*Kinosternon integrum*	Pr	LC	E

**Notes.**

Abbreviations for Endemic are E endemic, blank, non-endemic; for the Mexican protection Pr Special protection A Endangered, blank, no protection category; for the IUCN: LC, Least Concern NT Near Threatened VU Vulnerable EN Endangered

**Figure 3:**
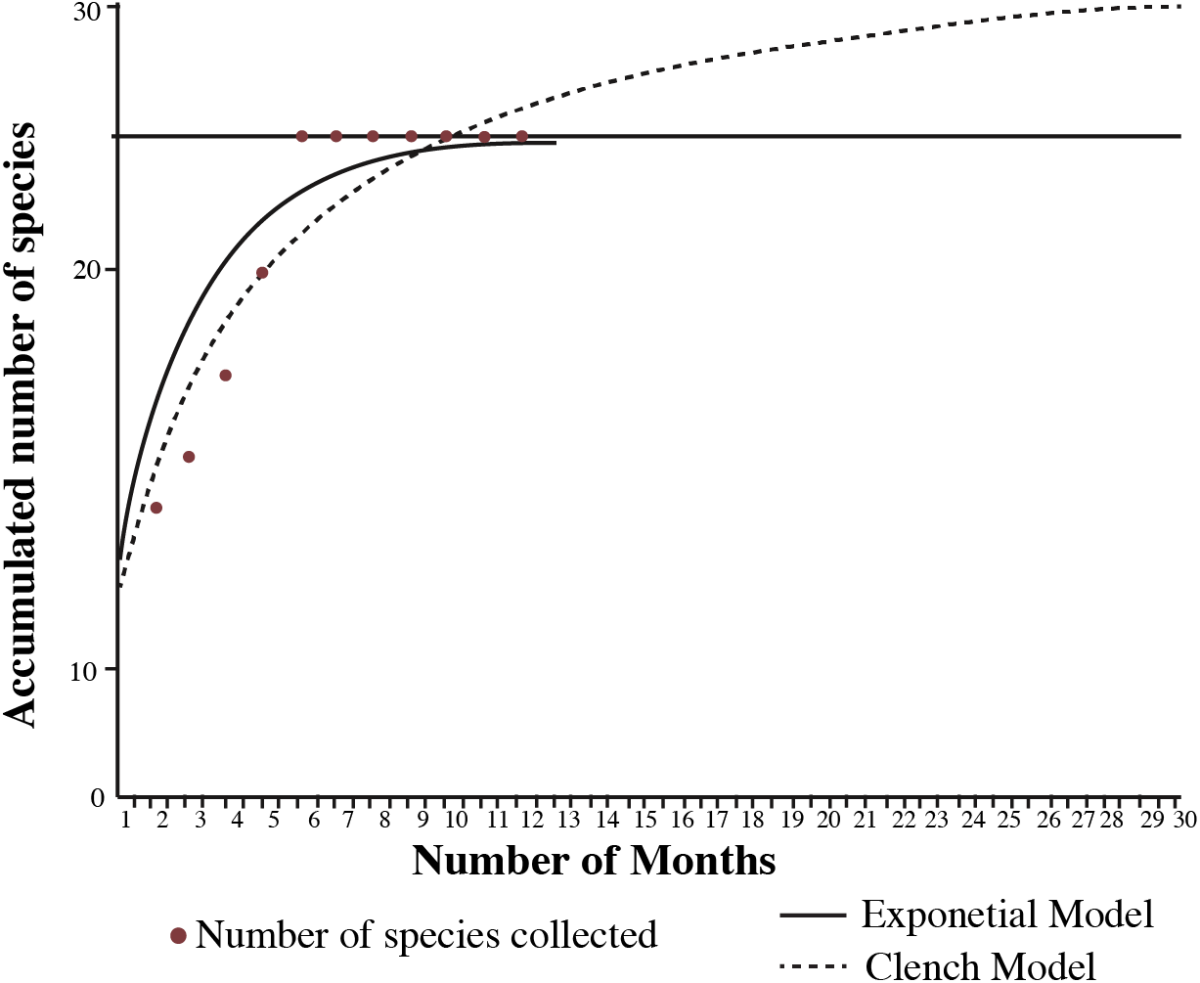
Species accumulation curve of amphibians and reptiles collected in the municipality of Nopala de Villagrán, Hidalgo, Mexico with the expected number of species from the Clench and Exponential model. Red dots are the number of species collected this study; the exponential model is the black line and the Clench model is the dashed line.

**Figure 4:**
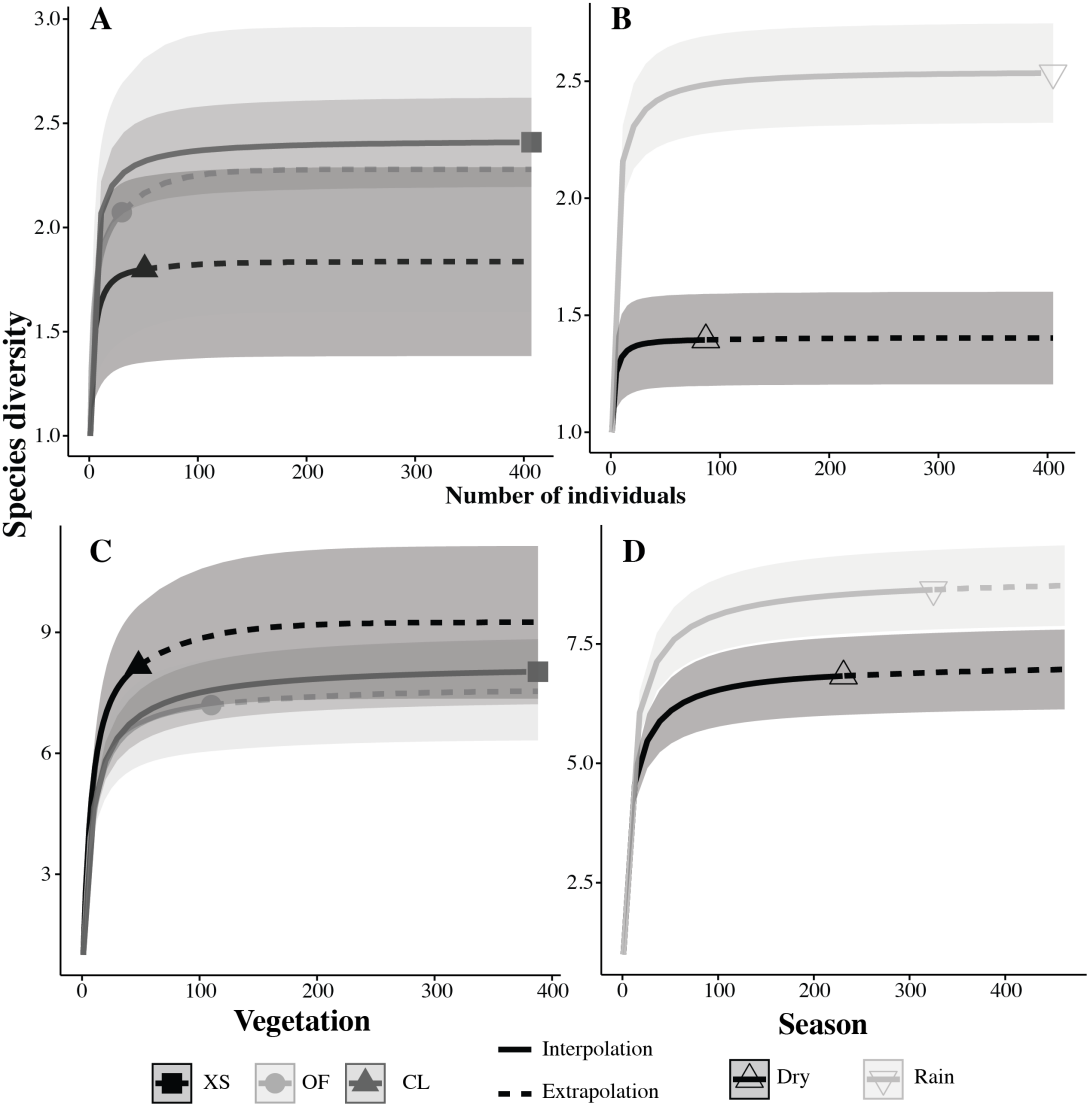
Individual based rarefaction curves for species of amphibians (A–B) and reptiles (C–D) by vegetation (A–C) and season (B–D) Vegetation types: XS, xeric scrub; OF, oak forest; CL, croplands.

The accumulation curve for sampling effort reached an asymptote in the sixth month, suggesting that the number of species encountered remained constant regardless of an increase in sampling effort. The exponential model expected the number of species collected in this study; while the Clench model suggested an additional six species could be encountered in the municipality if sampling effort was increased (Fig. 3). Furthermore, the sample coverage from the rarefactions curves was above 90%, indicating effective sampling of all vegetation types and seasons. Richness was higher for amphibians and reptiles in the rainy season with eight species exclusive to this season, while only two were exclusive to the dry season (Table 2). The higher richness in the rainy season was significant for both amphibians and reptiles, as shown by the non-overlapping 95% confidence intervals in the rarefaction curves (Fig. 4). As seen in such curves the difference was greater for amphibians than reptiles.

**Table 2:** Species list for the municipality of Nopala de Villagrán, Hidalgo, showing the number of individuals in each vegetation type, season and microhabitat.

Species^Rank−AbudanceCode^	Season		Vegetation			**Microhabitat**								
	D	R	MX	OF	CL	Ter	Sax	Arb	Scr	Rip	Aqu	Fos	DecT	Hum
*Hyla eximia*^**1**^	78	263	272	23	42	291	8		6		3	2	3	
*Hyla arenicolor*^**2**^	9	107	106	5	5	59	53		3		4			
*Lithobates montezumae*^**3**^		14	13	1		7				1	4			
*Spea multiplicata*^**4**^		16	12		4	16								
*Eleutherodactylus verrucipes*^**5**^		5	4	1		5								
*Sceloporus microlepidotus*^**6**^	71	57	92	29	8	50	19	38	6					5
*Sceloporus mucronatus*^**7**^	68	85	104	32	7	56	87	1						2
*Sceloporus spinosus*^**8**^	14	35	40	6	3	36	4	4						2
*Sceloporus torquatus*^**9**^	20	59	58	12	10	31	37	2						6
*Sceloporus scalaris*^**10**^	8			8		8								
*Aspidoscelis gularis*^**11**^	3		3			3								
*Conopsis lineata*^**12**^	24	17	29	6	2	37						2	1	
*Conopsis nasus*^**13**^	7	8	14	1		12						3		
*Thamnophis eques*^**14**^	3	20	10	6	6	21					1			
*Salvadora bairdi*^**15**^	2	6	7	3		8						2		
*Crotalus aquilus*^**16**^	4	2	4	2			3		2					
*Pituophis deppei*^**17**^		6	5		1	6								
*Thamnophis melanogaster*^**18**^		7	6		1	5					2			
*Coluber schotti*^**19**^	4	2			4	3								
*Lampropeltis ruthveni*^**20**^		2	2			1							1	
*Thamnophis cyrtopsis*^**21**^		2	2			2								
*Crotalus molossus*^**22**^	1	1	2			3								
*Kinosternon integrum*^**23**^	2	15	9	5	6	7					8			
*Kinosternon hirtipes*^**24**^		1	1			1								

**Notes.**

The microhabitats are Ter on the ground Sax saxicolous Arb arboreal Scr scrubby Rip riparian Aqu aquatic Fos fossorial DecT decaying trunks Hum human areas
Vegetation types XS xeric scrub OF oak forest CL croplands
Season R rainy D dry

The highest richness for both amphibians and reptiles was found in the xeric scrub, followed by the oak forest and croplands (Table 2). However, the differences in richness were not significant as both amphibian and reptile rarefaction curves showed great overlap in confidence intervals (Chao et al., 2014, Fig. 4). Additionally, the rarefaction curve for reptiles showed that when sampling is equalized the croplands could have higher richness than the xeric scrub vegetation (Fig. 4). This was not the case for amphibians in which the xeric scrub remained the richest vegetation (Fig. 4). In terms of endemic species the oak forest had proportionally more species endemic to Mexico with a Webb index of 2.75, it had more endemic than non-endemic. The xeric scrub had a Webb index of 1.2, indicating almost the same number of endemic to non-endemic. Finally, croplands had fewer endemic species that non-endemic ones as indicated by its Webb index of 0.85.

Vegetation types also varied in species composition, since seven species were restricted to only one type of vegetation. Five species of reptiles were restricted to the xeric scrub, but the only species from which we collected more than two individuals was the lizard *Aspidocelis gularis*. One species was restricted to the oak forest the lizard *Sceloporus scalaris*. Finally, the snake *Coluber schotti* was observed only in croplands (Table 2). Eight species showed a less restricted habitat and occupied two vegetation types. Five species were found in xeric scrub and oak forest, and three species were found in xeric scrub and croplands. Only nine species were distributed in all vegetation types, most of them being lizards (four species; Table 2). However, the Chao Jaccard similarity index showed that species composition was very similar for all pairwise comparison of vegetation types (Mean ± SE, MX-OF 0.91 ± 0.03, MX-CL 0.90 ± 0.03, OF-CL 0.81 ± 0.04).

In general, both amphibians and reptiles showed a preference for the terrestrial microhabitat, as all species were found there except the rattlesnake *C. aquilus* (Table 2; saxicolous and scrub habitats). The aquatic microhabitat was the second most commonly used by amphibians, while for reptiles it was the saxicolous microhabitat. The only microhabitat that had species exclusive to it was the terrestrial, with nine species (Table 2). However, four reptile species were represented by three or fewer individuals (*Aspidoscelis gularis, Thamnophis cyrtopsis, Crotalus molossus, Kinosternon hirtipes*). No amphibians were found in the arboreal microhabitat or human areas, and no reptiles were found in the riparian microhabitat.

**Figure 5:**
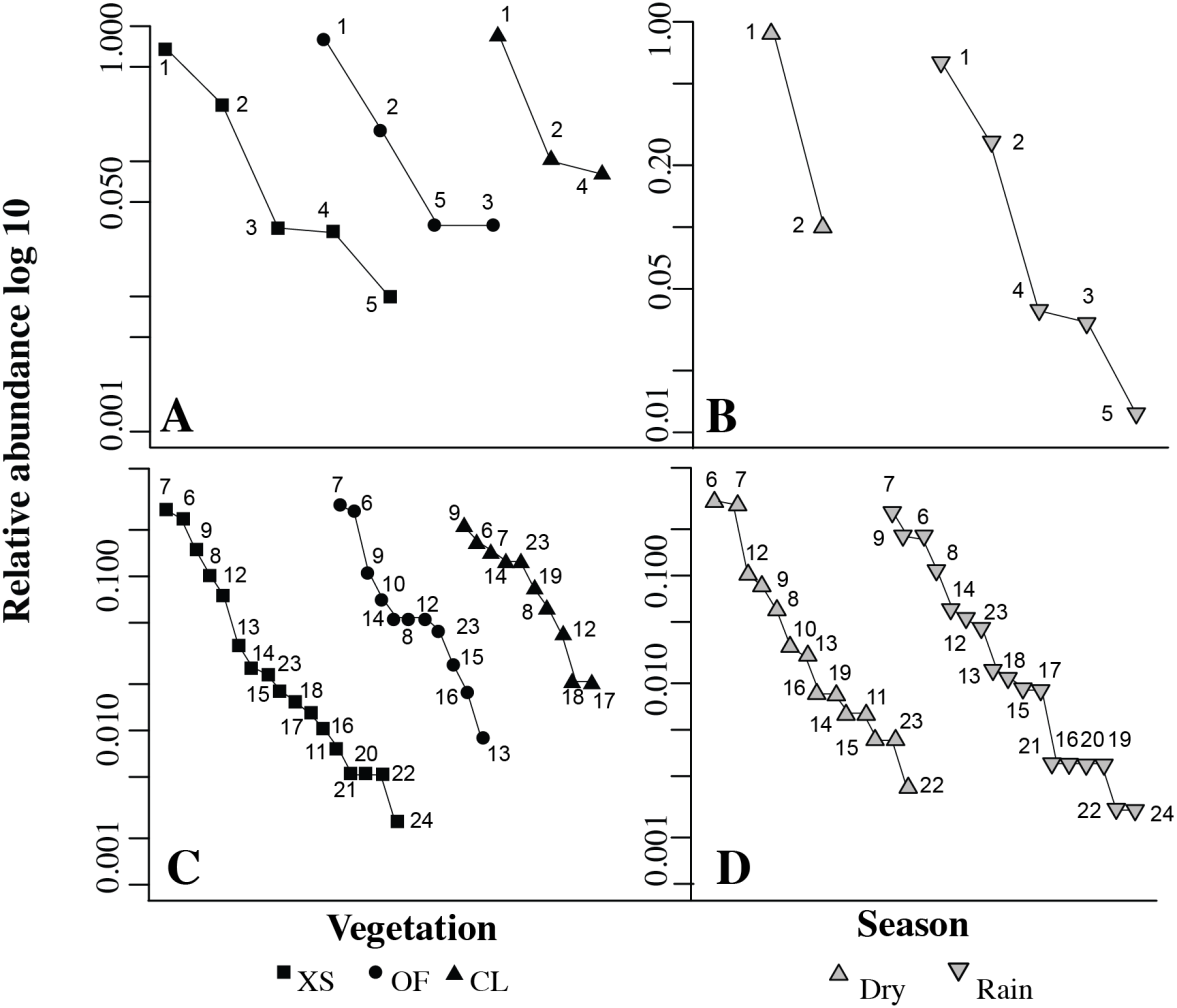
Rank abundance curves for species of amphibians (A–B) and reptiles (C–D) by vegetation (A–C) and season (B–D). Vegetation types: XS, xeric scrub; OF, oak forest; CL, croplands. Rank abundance codes can be found in Table 2.

The rainy season was not only the richest in the number of species of amphibians and reptiles but also in abundance, as the number of individuals found was at least twice that observed in the dry season. The rank-abundance curves for amphibians show that overall two species are the most abundant in the municipality regardless of vegetation and season, the frog *Dryophytes eximia* and the frog *Dryophytes arenicolor* (Fig. 5). Although as mentioned above, the number of individuals for this two species did increase in the rainy season (Table 2). For reptiles the rank abundance curve showed different patterns for vegetation and season. However, it is interesting that although they are subtle differences, three species of the Spiny lizard (*Sceloporus*) were always present in the higher abundances, suggesting that they are dominant in all vegetation types and seasons. Subtle differences existed in the order of dominance of this three species for each vegetation type and the displacement of the lizard *S. torquatus* to fourth rank by the snake *Conopsis lineata* in the dry season (Fig. 5).

## Discussion

The herpetofaunal richness recorded for the study area is relatively high, since most of the other semi-arid regions surveyed in the state of Hidalgo report a lower number of species (Camarillo & Casas-Andreu, 1998 (Zacualtipán and Huayacocotla); Hernández Pérez, 1998 (Metztitlán); Gómez Mendoza, 2007 (Tepeji del Río de Ocampo)), while those that report more species have covered much larger areas (Mendoza-Quijano, 1990; Camarillo & Casas-Andreu, 2001; Huitzil Mendoza, 2007; Hernández Salinas, 2009; Vite-Silva, Ramírez-Bautista & Hernández-Salinas, 2010). This means that our study reports higher richness for a considerably smaller area. For example, only 31 species have been reported in the section of the Trans-Mexican Volcanic Belt that crosses Hidalgo (Flores-Villela & Canseco-Márquez, 2007), but the municipality of Nopala de Villagrán alone has almost the same number of species (24) in a much smaller area. In addition, we found a species previously unreported for Hidalgo, the snake *Lampropeltis ruthveni* (Roth-Monzón Flores-Villela, Mendoza-Hernández & Flores-Villela, 2011).

According to the exponential model of the species accumulation curve and the sample coverage from the rarefaction curves, our sampling for the municipality in all vegetation types and seasons can be scored as “good”. However, the Clench model indicates that more species could have been collected with increased sampling effort, since the curve is not asymptotic. These likely included the lizard *Sceloporus parvus,* which was collected just outside the municipality’s limits, and the Mole salamander (*Ambystoma* sp.), which was observed during the fieldwork though no voucher was obtained for species identification (probably *A. velasci*). Another species that is probably present is the lizard *Phrynosoma orbiculare,* which has been mentioned by the local people of the town of Nopala. Other species potentially occurring in the study area due to geographic proximity are: the lizard *Barisia imbricata,* the coral snake *Micrurus tener* and the rattlesnake *Crotalus polystictus* (Matias Ferrer, 2004; Campos Rodríguez, 2005; Zaldívar-Riverón, NietoMontesde Oca & Laclette, 2005; Ramírez-Bautista et al., 2014).

Our rarefaction curves show that there were no significant differences among vegetation for both amphibians and reptile richness (Chao et al., 2014). Nevertheless, for reptiles the rarefaction curves indicated that if sampling was equalized, croplands would have had a higher richness (Fig. 4). This implies that for reptiles our study conforms on croplands being species rich, probably because of the multitude of microhabitats they can provide and sources of food as has been found on other studies (Martínez-Castellanos, 1994; Salazar-Arenas, 2001; González-Hernández & Garza-Castro, 2006; Fernández-Badillo & Mayer-Goyenechea, 2010). This was not the case for amphibians in which xeric scrub was indicated by the rarefaction curves to have the higher species richness (Fig. 4). The pattern may not hold for amphibians due to aggressive farming practice in the municipality (i.e., fertilizers and heavy machinery are used, there is extensive clearing of the vegetation cover and cyclic burning). Such differences in management and type of crop have been reported to cause differences in species richness (Salazar-Arenas, 2001; González-Hernández & Garza-Castro, 2006) and amphibians have been reported to be more sensitive to such conditions (Brühl et al., 2013; Böll, et al., 2013).

The lack of significant differences between vegetation types for both amphibians and reptiles could be due to a homogenous distribution of the amphibians and reptiles in the municipality. Given that the Jaccard- Chao similarity index among vegetation for species richness was above 80%, indicating that the vegetation types shared several species. Although unique species were found for all vegetation types, those found in the xeric scrub could be unique to this vegetation due to their low abundance (less than three individuals). Nevertheless, in both croplands and oak forest a unique species was found for which low abundance did not seem to be the reason for the resulting pattern. Additionally, oak forest and croplands had the lowest similarity (80%). This can be related to unique microenvironmental characteristics of each vegetation type (Atauri & Lucio, 2001; Tews, Brose & Jeltsch, 2003; Martins, Proença & Pereira, 2014)) or to a lack of ability to acclimate or access the different vegetations, as has been shown in the case of the lizard *Sceloporus scalaris* which may be affected by goat grazing and thus likely avoids such human impacted areas (Bock, Smith & Bock, 1990). An increase in human activity as seen in the area could have implications for this non-share species.

Furthermore, the oak forest had the highest proportion of endemic species as indicated by the Webb index (2.75). Oak forest is known for its importance for endemic vertebrates in Mexico (Flores-Villela & Gerez, 1994; Canseco-Márquez, 1996), for which its loss can have conservational implications. Nevertheless, the Jaccard-Chao similarity index for the xeric scrub with both the oak forest and croplands was high (90%), meaning that the xeric scrub may support almost all current species in the municipality.

Our data show that seasonality is an important factor in the richness of amphibians and reptiles. The rarefaction plots showed that the differences in richness were significant and higher for the rainy season for both taxonomic groups (Fig. 4). Furthermore, during the rainy season, amphibians were able to enter areas disturbed by human activity, such as roads and cattle pastures, a behavior that has been observed previously in other amphibians (Santos-Barrera & Flores-Villela, 2004). Other studies have suggested that reptile distributions are constrained by temperature and amphibian by humidity (Navas, 2002; Buckley, Hurlbert & Jetz, 2012; Chen, 2013; Ortiz-Yusty, Páez & Zapata, 2013). This pattern is matched by amphibians in Nopala de Villagrán, but not by reptiles, since mean temperature by season does not vary greatly (14.5 to 14.7 °C; INEGI, 2002). However, it is possible that reptile distributions may be restricted by food availability. Differences in prey availability have been found in previous studies (Luiselli, 2006; Whitfield & Donnelly, 2006; García, 2008), making the dry season an ideal time for insects and the rainy season for amphibians and fishes, in such way that changes in food items could be the reason for the reptile distribution pattern found in the municipality. This hypothesis should be tested, as the present study did not directly measure food preference. Nonetheless, it is based on the different species found in the municipality, in which more lizard species were found in the dry season, while more snake species were found in the rainy season. This pattern seems to match food habits, as lizards are known to feed on invertebrates more often than snakes (Vitt et al., 2003; Shine & Wall, 2007).

Microhabitat use for amphibians also suggested humidity is a limiting factor, since amphibians were found active when condition permitted or in water related microhabitats (aquatic and riparian; Table 2). A few individuals of the frog *Dryophytes eximia* were found in fossorial and decaying trunks, these are not considered typical microhabitats for this species since they have not been reported in other general accounts of *D. eximia* (Duellman, 2001; Hammerson & Canseco-Márquez, 2010; AmphibianWeb, 2013). This species may seek these microhabitats to protect itself from dehydration, and if so this microhabitat may be important to consider for future inventories, especially in drylands.

Almost all of the reptile species preferred on the terrestrial microhabitat, which is not unusual since they were found basking on the ground or hiding under rocks. However, those that were found utilizing only one microhabitat were also found in one vegetation type and most of them were also the least abundant (Table 2; Fig. 5). This may suggest that such vegetation unique microenvironment restricts their distribution or that their low abundance is why they where found in such restrictive conditions (Fig. 5). The one species of reptile found in an unusual microhabitat was the rattlesnake *Crotalus aquilus* as it is usually found in rocky or grassland areas (Armstrong & Murphy, 1979; Campbell & Lamar, 2004; Meik, Mociño Deloya & Setser, 2007). However, during our fieldwork it was found basking on agave plants at a height of approximately one meter. This association with agave plants is known to the IUCN (Mendoza-Quijano & Quintero Díaz, 2007), and there are other observations on rattlesnakes (*C. triseriatus)* a meter above the ground (Rebón-Gallardo & Flores-Villela, 2015), thus this behavior may be a more common than currently thought. The use of agaves by this snake could be an important aspect to consider in the future, especially in relation to conservation, since agave is an economically important resource in Mexico, particularly in semi-arid regions like the municipality of Nopala de Villagrán (CONABIO, 2005; García-Herrera, Méndez-Gallegos & Talavera-Magaña 2010).

Most of the species were not found in human areas, except for four species of Spiny lizards (*Sceloporus*). These four Spiny lizards may be generalists capable of adapting to disturbed habitats, given that they were usually dominant according to our rank-abundance curves in all the vegetation types and seasons (Fig. 5; see dry season for exception). This type of behavior has been reported for at least one of these species (*Sceloporus microlepidotus*; Vega-López & Álvarez, 1992; Ramírez-Bautista et al., 2009). Likewise, all other species of amphibians that were among the highest rank in relative abundance (*Dryophytes eximia* and *D. arenicolor*; Fig. 5) were found in all vegetation types and seasons, which may mean they are better able to withstand the seasonal changes in temperature and humidity.

The species reported in this study fill an important geographic gap in our knowledge of amphibian and reptile diversity in the state of Hidalgo, Mexico. The information obtained may be of use in developing a management program for the municipal reserve of Cerro Nopala that was decreed, and thus help preserve amphibians, reptiles and other animal groups. Clearly, additional studies are necessary to better understand the habits of amphibians and reptiles in semi-arid regions, and the impact of humans on them, since these often ignored areas shelter a considerable diversity of amphibians and reptiles.

##  Supplemental Information

10.7717/peerj.4202/supp-1 Appendix S1 Species list with MZFC accession numbers for each individual collected

## Additional Information and Declarations

### Field Study Permissions

The following information was supplied relating to field study approvals (i.e., approving body and any reference numbers): Collecting permits were issued by the Secretaría de Medio Ambiente y Recursos Naturales (permit # FAUT-0015).

### Data Availability

The following information was supplied regarding data availability: The data is included in Table 2.
