# Supplementary material for: Amphibian and reptile biodiversity in the semi-arid region of the municipality of Nopala de Villagrán, Hidalgo, Mexico
Source: PeerJ. 2018 Jan 4;6:e4202. doi: 10.7717/peerj.4202 (PMC5756618; doi:10.7717/peerj.4202)
Supplement: Appendix S1 [file peerj-06-4202-s001.docx]

**Appendix 1. Species list with MZFC accession number for each individual collected.**

| **Species** | **MZFC Accession Number** |
| --- | --- |
| *Dryophytes eximia* | 33263-33269, 32295, 32298, 32324, 32327, 32331, 32368-32370, 32384, 32385, 32387 |
| *Dryophytes arenicolor* | 32283, 32296, 32297, 32304, 32323 |
| *Lithobates montezumae* | 32322, 32336-32340, 32346, 32349, 32350, 32352, 32353, 32361 |
| *Spea multiplicata* | 32301, 32315, 32316, 32335 |
| *Eleutherodactylus verrucipes* | 32312, 32313, 32341, 32358 |
| *Sceloporus microlepidotus* | 32388, 32271-32273, 32276, 32277, 32282, 32285, 32299, 32365 |
| *Sceloporus mucronatus* | 32279, 32294, 32307, 32325, 32328, 32342, 32347, 32354, 32356, 32379-32381 |
| *Sceloporus spinosus* | 32284, 32286, 32291, 32293, 32310, 32311, 32332, 32333, 32351, 32374, 32394 |
| *Sceloporus torquatus* | 32275, 32278, 32280, 32281, 32387, 32300, 32344, 32345, 32348, 32364 |
| *Sceloporus scalaris* | 32270, 32274, 32373, 32375, 32382 |
| *Aspidoscelis gularis* | 32292 |
| *Conopsis lineata* | 32389-32391, 32403-32406, 32411, 32408, 32409 |
| *Conopsis nasus* | 32314, 32320, 32326, 32330, 32372, 32377 |
| *Thamnophis eques* | 32302, 32303, 32308, 32321, 32329, 32378, 32383 |
| *Salvadora bairdi* | 32319, 32343, 32355, 32359, 32366 |
| *Crotalus aquilus* | 32392, 32401, 32402, 32407, 32410 |
| *Pituophis deppei* | 32412, 32334, 32360, 32363, 32367, 32371 |
| *Thamnophis melanogaster* | 32305, 32306, 32309, 32318 |
| *Coluber schotti* | 32362, 32376 |
| *Lampropeltis ruthveni* | 23645, 23646 |
| *Thamnophis cyrtopsis* | 32317, 32357 |
| *Crotalus molossus* | 32386 |
| *Kinosternon integrum* | 32393, 32395, 32396, 32398, 32399, 32400 |
| *Kinosternon hirtipes* | Photograph voucher |
